# Supplementary material for: The Evolution and Characterization of the RNA Interference Pathways in Lophotrochozoa
Source: Genome Biol Evol. 2024 May 7;16(5):evae098. doi: 10.1093/gbe/evae098 (PMC11114477; doi:10.1093/gbe/evae098)
Supplement: evae098_Supplementary_Data [file evae098_supplementary_data.zip › Supplementary_materials.zip!/selected_miRNAs/NoGe_selected_miRNAs/known_miRNA_families_pdf_files/NMRB01001262.1_Notospermus_geniculatus_strain_Ushimado_scaffold1262,_whole_genome_shotgun_sequence_26853.pdf]

Provisional ID	:	NMRB01001262.1_Notospermus_geniculatus_strain_Ushimado_scaffold1262,_whole_genome_shotgun_sequence_26853
Score total	:	245011.5
Score for star read(s)	:	3.9
Score for read counts	:	245002.7
Score for mfe	:	1.9
Score for randfold	:	0
Score for cons. seed	:	3
Total read count	:	480574
Mature read count	:	471927
Loop read count	:	16
Star read count	:	8631

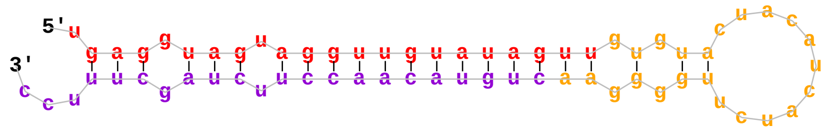

Genome coordinates: NMRB01001262.1 36732-36799

pre-miRNA sequence:
UGAGGUAGUAGGUUGUAUAGUUUGAGGUAGUAGGUUGUAUAGUUGUGUACUACAUCAUCUUGGGGAACUGUACAACCUUCUAGCUUUCC

Mature sequence: UGAGGUAGUAGGUUGUAUAGUU

Aligned mature sequence of the *N. geniculatus* pre-miRNA with the top 5 hits from the BLAST search against the pre-miRNA mirGeneDB set:

```
                            **********************
Nge_Let-7                   ugagguaguagguuguauaguu
Csc-Let-7-P18b_5p           ugagguaguagguuguauaguu
Lgi-Let-7_5p                ugagguaguagguuguauaguu
Lpo-Let-7-P17_5p            ugagguaguagguuguauaguu
Bge-Let-7_5p                ugagguaguagguuguauaguu
Lan-Let-7_5p                ugagguaguagguuguauaguu
```
